# *pyResearchInsights*—An open‐source Python package for scientific text analysis

**DOI:** 10.1002/ece3.8098

**Published:** 2021-09-17

**Authors:** Sarthak J. Shetty, Vijay Ramesh

**Affiliations:** ^1^ Center for Ecological Sciences Indian Institute of Science Bengaluru India; ^2^ Department of Ecology, Evolution and Environmental Biology Columbia University New York NY USA

**Keywords:** automated content analysis, exploratory analysis, natural language processing

## Abstract

1. With an increasing number of scientific articles published each year, there is a need to synthesize and obtain insights across ever‐growing volumes of literature. Here, we present *pyResearchInsights*, a novel open‐source automated content analysis package that can be used to analyze scientific abstracts within a natural language processing framework.
2. The package collects abstracts from scientific repositories, identifies topics of research discussed in these abstracts, and presents interactive concept maps to visualize these research topics. To showcase the utilities of this package, we present two examples, specific to the field of ecology and conservation biology.
3. First, we demonstrate the end‐to‐end functionality of the package by presenting topics of research discussed in 1,131 abstracts pertaining to birds of the Tropical Andes. Our results suggest that a large proportion of avian research in this biodiversity hotspot pertains to species distributions, climate change, and plant ecology.
4. Second, we retrieved and analyzed 22,561 abstracts across eight journals in the field of conservation biology to identify twelve global topics of conservation research. Our analysis shows that conservation policy and landscape ecology are focal topics of research. We further examined how these conservation‐associated research topics varied across five biodiversity hotspots.
5. Lastly, we compared the utilities of this package with existing tools that carry out automated content analysis, and we show that our open‐source package has wider functionality and provides end‐to‐end utilities that seldom exist across other tools.

## Graphical abstract

With an increasing number of scientific articles being published each year, there is a need to synthesize and obtain insights across large volumes of literature. Hence, we developed *pyResearchInsights*, a novel open‐source, modular, end‐to‐end automated content analysis package that can be used to analyze scientific abstracts within a natural language processing framework.

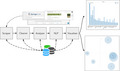

## 1 INTRODUCTION

Keeping track of conceptual and methodological developments in any scientific discipline is imperative to advance research. An exponential growth in published scientific literature has made it extremely difficult to keep track of scientific advancements (Roll et al., 2018). Within the field of ecology, we have observed a twofold increase in published literature over the last decade (Nunez‐Mir et al., 2016). As the volume of academic literature grows rapidly each year, it is difficult to methodically analyze and synthesize the extent of knowledge on multiple topics (Ferreira et al., 2016; Tejeda‐Lorente et al., 2014). Hence, there arises a need for automated tools that can analyze large volumes of academic literature.

Automated content analysis (ACA) tools are a suite of statistical analysis tools that can be used to identify the thematic composition of large volumes of text (Boumans & Trilling, 2016; Nunez‐Mir et al., 2016; Stockwell et al., 2009). Valuable insights with respect to the overall frequency of words and their relationships with other words, along with broad topics of research, can be ascertained using ACA tools and thereby serve as a crucial aid during the preliminary stages of exploratory research (Nunez‐Mir et al., 2016). For example, Fisher et al. (2011) used a combination of text analysis and Google Maps to analyze the spatial coverage of coral reef research. Similarly, Dyer (2015) used text mining and natural language processing to examine the content of manuscripts associated with the field of landscape genetics. Existing ACA tools are available on a subscription basis and have been used across publications ranging from topics of research in disease ecology and forestry research to analysis of global trends in ecological research (Han & Ostfeld, 2019; Nunez‐Mir et al., 2016; McCallen et al., 2019). However, many of these tools are expensive and inaccessible to a large portion of the scientific community, especially those from the global south (Reidpath & Allotey, 2020).

**FIGURE 1:**
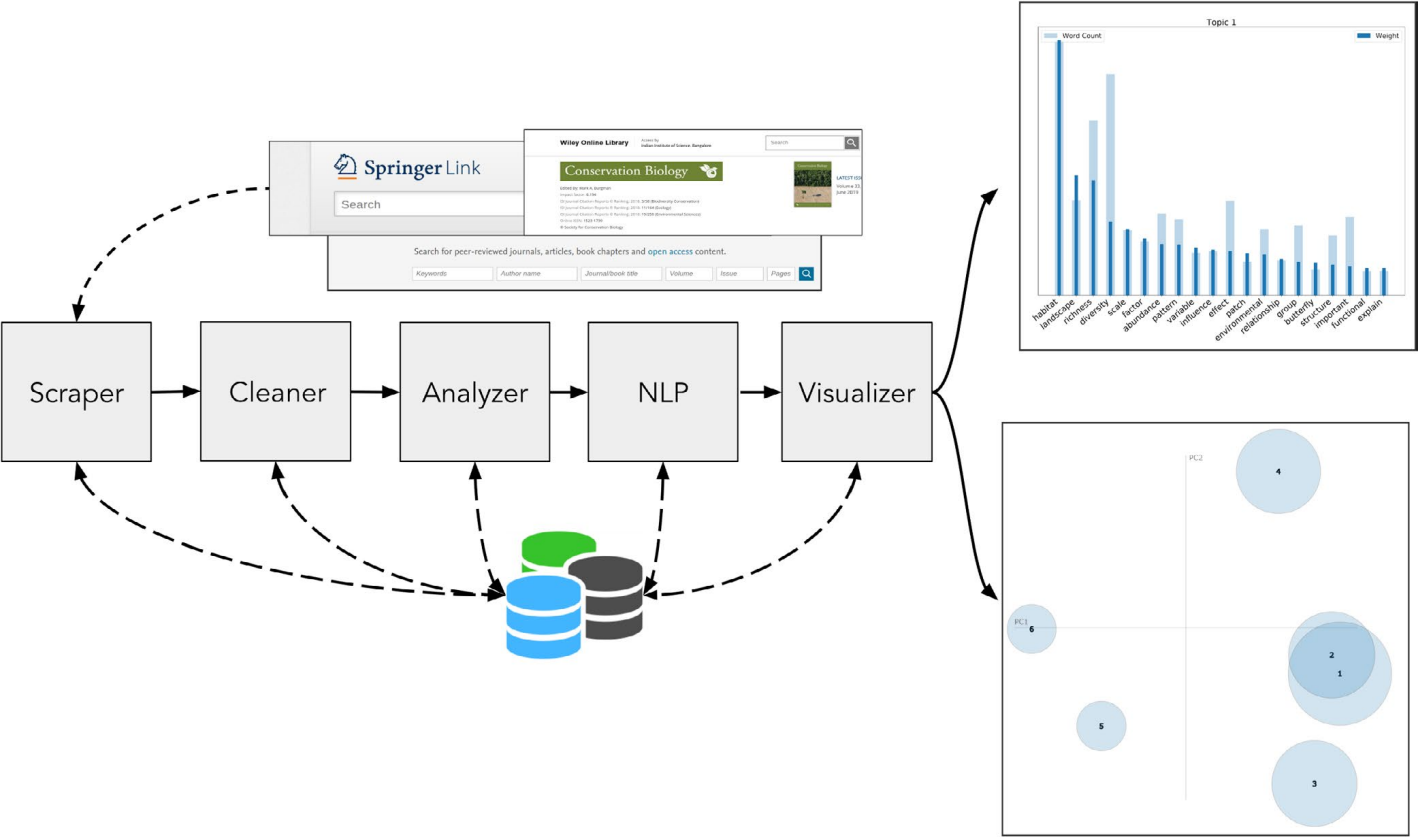
A schematic of the *pyResearchInsights* pipeline is shown here. **Scraper**: This component collects publication abstracts from Springer containing the search words provided by the user. The user input to the Scraper is the search words to be queried from Springer. **Cleaner**: This component cleans the corpus of text retrieved and rids it of special characters and poor formatting. The input to the Cleaner is the raw abstracts collected by the Scraper. **Analyzer**: This component measures the temporal frequency of words occurring across the abstracts database. The input to the Analyzer is the cleaned abstracts generated by the Cleaner. **NLP Engine**: This component trains an LDA model using the words within the abstracts collected to identify research topics. The user can provide their own text files as input to the NLP Engine and analyze research topics, instead of relying on the Scraper to generate these text files. **Visualizer**: This component presents the topic modeling results in the form of LDA visualizations and frequency and weight charts. The input to the Visualizer is the cleaned, tokenized abstracts from the NLP Engine. **Note:** The LDA charts and bar plots presented above are for representation purposes only

Here, we present *pyResearchInsights*, an open‐source end‐to‐end automated content analysis package, that (a) collects scientific abstracts, (b) cleans the texts collected, (c) performs ACA, and (d) presents interactive visualizations. Our package consists of five modular components: a Scraper (to collect published scientific abstracts, given a set of search words), a Cleaner (to get rid of formatting errors), an Analyzer (to measure the frequency of specific words), a natural language processing (NLP) engine (to perform topic modeling on the abstracts), and a Visualizer (to present the topic modeling results) (Figure 1). While ACA can be performed with existing packages, there is a lack of a truly open‐source end‐to‐end tool that can analyze scientific texts in this manner, without wrangling with and integrating multiple libraries and dependencies. The functionality of the package and each of its modules is presented in the case studies below.

## 2 CASE STUDY A: TOPICS OF AVIAN RESEARCH IN THE TROPICAL ANDES

In this case study, we illustrate the components of the *pyResearchInsights* pipeline by showcasing topics of research pertaining to a specific geographic area. We chose the example of avian research in the Tropical Andes because of the high diversity of bird species documented in the Neotropics, especially along the mountain ranges of the Andes (Quintero & Jetz, 2018). Since *pyResearchInsights* is open‐sourced and made available on *pip*, users can run the package on modern cloud environments such as Google Colab and Jupyter, leveraging powerful computational resources available on these platforms. In order to demonstrate this capability of *pyResearchInsights* ‐‐ the retrieval of publications, analysis, and visualization of topics presented in this case study was carried out entirely on a Google Colab notebook that can be accessed here: https://colab.research.google.com/drive/1gZjOKr5pfwVMuxCSaGYw20ldFpV4gVws?usp=sharing. We advise users to utilize the package in a Google Colab environment to ensure there are no dependency conflicts while installing *pyResearchInsights* on their local machines. Advanced users, however, can install the package on their local machines as well.

The process of ACA begins by supplying a list of search words. Here, we supplied the search words—“Tropical Andes Birds” to the **Scraper** component of the package, which uses the search words to collect a set of 1,131 abstracts from *Springer* (https://link.springer.com/). The Scraper collects only journal abstracts from Springer containing the exact search words provided by the user. Users can also query search words as Boolean strings in order to return abstracts containing specific combinations of search words. The Scraper component is solely an interface between the user and *Springer* for retrieving papers, analysis, and topic modeling. For example, if the search words “Tropical Andes Birds” are keyed into the package for abstract retrieval, abstracts containing all three words, “tropical”, “andes,” and “birds,” are retrieved for analysis by *pyResearchInsights*.

This module utilizes the Beautiful Soup (Richardson, 2007) and Urllib (urllib, 2008) open‐source Python web‐scraping packages to interface with *Springer*. In addition, the Scraper collects only publicly available data such as abstracts, author names, and date of publication (Fiesler et al., 2020). Furthermore, the data collected are not redistributed and used solely for the purposes of topic modeling and exploratory visualization.





searchwords = “Tropical Andes Birds”



abstracts_log_name, status_logger_name =


pre_processing(searchwords)



scraper_main(searchwords, abstracts_log_name, status_logger_name






abstracts_log_name is a time‐stamped folder that contains all the abstracts scraped during the session. The status_logger logs the functions executed during the code run for debugging in case of early termination of the code.

The **Cleaner** is then used to remove stop words, special characters, and symbols from the abstracts. The NLTK library (Bird et al., 2009) in Python has a list of stop words that are used to prune the corpus collected by the Scraper. This list of stop words can be expanded as well as we have done in Line 48 of the Cleaner script to improve the quality of the topic modeling results. Through analysis of the data retrieved from the Scraper, we noticed that the special characters that appear in texts collected by the package appear predominantly with the forward slash (“\”). Hence, we specifically searched for this special character while cleaning the corpus and got rid of strings containing it to ensure a clean corpus. The user can remove additional special characters from the corpus collected by modifying the dirty_elements list in Line 16 of the Cleaner.py script of the package. Consider the word “black\‐backed,” which is treated as a single word (since there's a hyphen connected “black\” and “backed”). When the Cleaner encounters the “\” while analyzing “black\‐backed” it removes the word from the corpus. The user can tweak this cleaning operation in Line 59 of the Cleaner script. Such errors occur due to poor formatting in manuscripts and do not hamper the performance of the NLP Engine significantly.





cleaner_main(abstracts_log_name, status_logger_name)






The cleaned corpus is passed through the **Analyzer** that generates a Pandas (McKinney, 2010) DataFrame to store the frequency of occurrence of various words in the abstracts in a.CSV file, which can be utilized by the user for further analysis. This DataFrame is not necessarily utilized by other modules of *pyResearchInsights*.





analyzer_main(abstracts_log_name, status_logger_name)






The cleaned corpus of text is also provided to the **NLP Engine**, which utilizes gensim (Řehůřek, & Sojka, 2010) and spaCy (Honnibal & Johnson, 2015) packages to train a Latent Dirichlet Allocation (LDA) language model (Blei, 2012; Blei et al., 2003) on bigrams and trigrams generated from the corpus (Tan et al., 2002 and Lafferty et al., 1992). An n‐gram is essentially a sequence of n‐words from a given sample of text. For example, consider the text “Tropical Andes is a biodiversity hotspot.” This text can be broken down into unigrams such as “tropical,” “andes,” “biodiversity,” or bigrams such as “tropical andes,” “biodiversity hotspot,” or trigrams such as “tropical andes biodiversity” or more.

First, the LDA model breaks the cleaned corpus of text into n‐grams as described above. Second, the model analyzes the frequency of each n‐gram word present in the corpus and assigns a weight. This weight is assigned on the basis of the frequency of occurrence of an n‐gram word and its co‐occurrence with other n‐gram words (Blei et al., 2003). During this weight assignment process, certain combinations of n‐gram words display high semantic associations (i.e., those words that closely occur together throughout the corpus of text). Such words are clustered together by the LDA model under the same topic, whereas those combinations of words that display poor semantic associations are weeded out (Blei et al., 2003). In addition, commonly used stop words are also removed from the corpus by the gensim package. This list of stop words can be modified by the user in the NLP Engine script. The user can also vary the number of topics generated by passing an argument to the NLP Engine, as shown below.





nlp_engine_main(abstracts_log_name, num_topics = 12,


status_logger_name)






**FIGURE 2:**
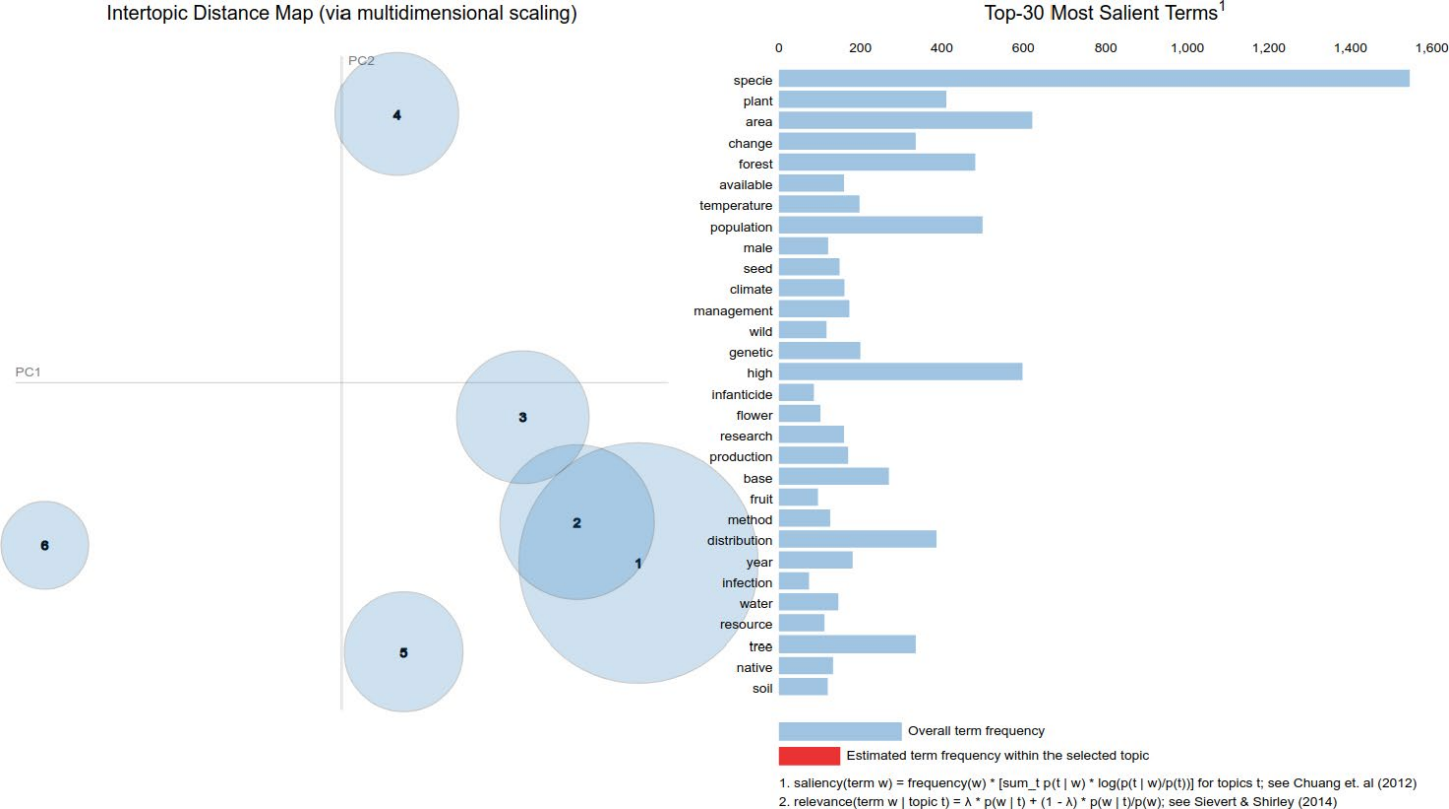
The topics are presented using interactive concept maps as shown here. Topics are represented as circles on the left‐hand panel of the *pyLDAvis* visualization (intertopic distance map), where the area of the topic circles is proportional to their relative prevalence in the corpus (Sievert & Shirley, 2014; Mabey, 2015). On the right‐hand side of the *pyLDAvis* visualization are the words belonging to various topics. Clicking a word from this panel presents the distribution of that word among the topics. Clicking on a topic from the left‐hand panel presents the words belonging to that topic. When a sufficiently large corpus of text is provided to the LDA Model, the first few topics generated are general ideas discussed across the documents in the corpus

**FIGURE 3:**
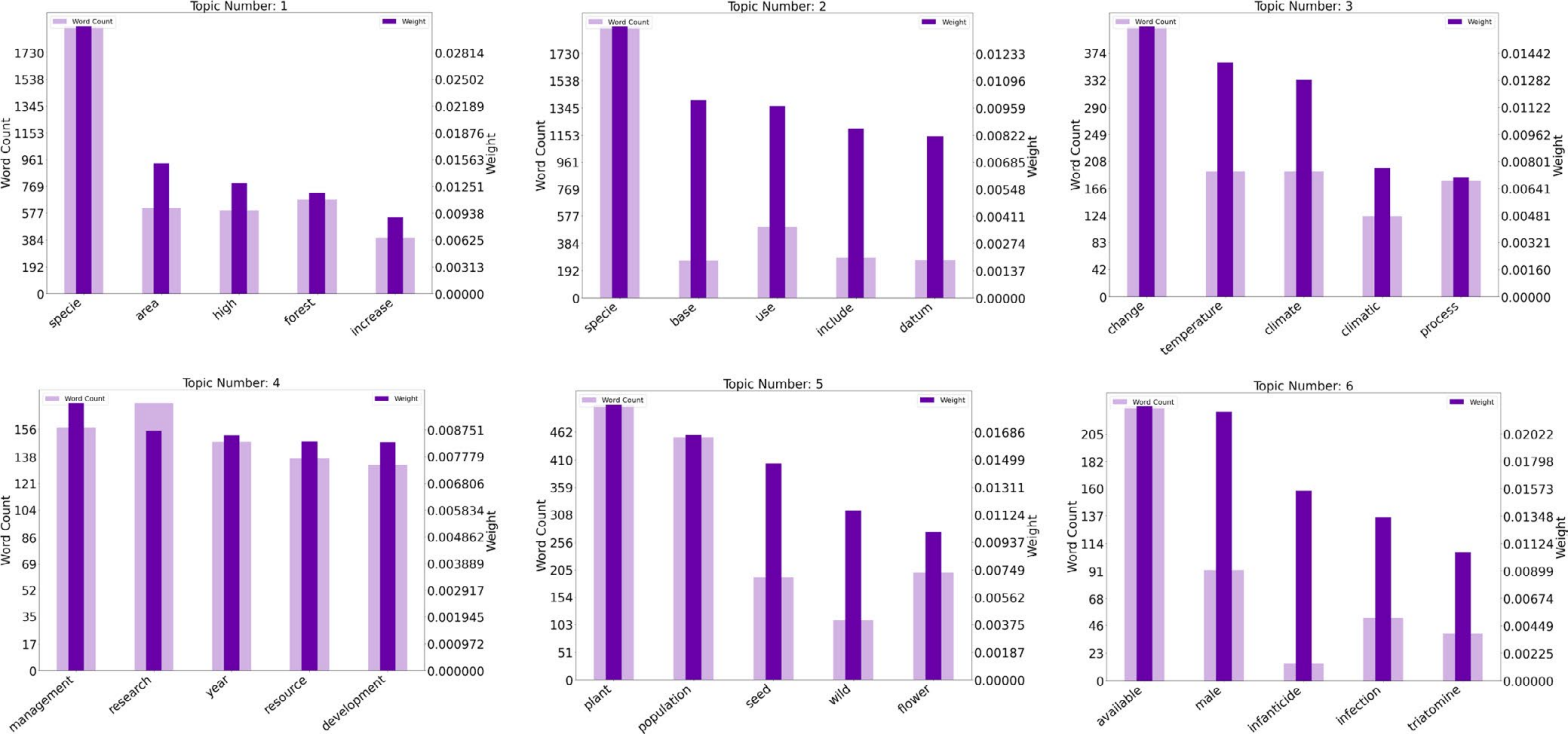
The Visualizer also generates word count and weight charts for each topic. The Y‐axes on the left and right represent the word count and weight, respectively, for each of the words on the X‐axis

Finally, this LDA model is passed to the **Visualizer** which generates a pyLDAvis (Mabey, 2015) visualization of the topic clusters (Figure 2). The user can now make a formal inference of the topic clusters by analyzing the frequency and weights of words within a topic. However, the assignment of topic labels by computer systems is an open‐ended research question in the field of natural language processing (Han Lau et al., 2011). Users can utilize their domain knowledge to assign labels to each topic by analyzing the words under each topic (Khandkar, 2009). For instance, exploratory analysis of the words and the topics of avian research across the Tropical Andes suggests that a number of publications are associated with climate change (Topic 3) and plant ecology (Topic 5) (Figure 2 and Figure 3).

## 3 CASE STUDY B: TOPICS OF CONSERVATION RESEARCH ACROSS BIODIVERSITY HOTSPOTS

In this case study, we used *pyResearchInsights* to identify topics of research within the broad domain of conservation biology and analyzed how these conservation‐associated research topics varied across biodiversity hotspots.

A total of 22,561 abstracts were downloaded (as of July 2019) from eight journals in the field of conservation biology, which span a wide range of literature associated with conservation biology (Table 1). We hypothesize that published literature in these journals will be representative of global conservation research topics. We used the **Cleaner** and **Analyzer** to remove special characters and obtain the frequency of words, respectively, across the abstracts.

**TABLE 1:** Conservation science journals and number of abstracts scrapped

Journal Name	Number of Abstracts
Conservation Biology	4,991
Biological Conservation	5,073
Biodiversity and Conservation	4,811
Environmental Conservation	5,350
Global Ecology and Conservation	730
Journal for Nature Conservation	789
Perspectives in Ecology and Conservation	95
Conservation Letters	722
Total	22,561

Note

To collect the corpus of abstracts for this case study, the authors utilized Columbia University's subscription to the 8 conservation journals mentioned since these abstract data are not publicly available. A total of 22,561 abstracts were collected across these 8 journals as of July 2019.

**FIGURE 4:**
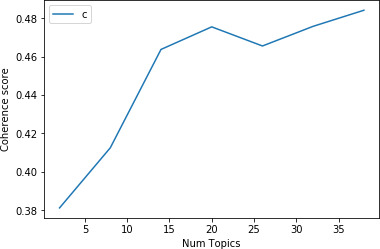
From our analysis, we found that the coherence score for our model on our dataset peaks at around 12 topics, as observed in the above chart. Beyond 12 topics, the improvement to the coherence score of the model is imperceptible and leads to loss of coherence between topics

Using the **NLP Engine,** an LDA model was trained on these abstracts to generate 12 topics of global conservation research. The clustering of topics by language models is judged on the basis of “coherence” scores (He et al., 2008). A topic is said to be coherent if the words within the topic are semantically similar to each other. We calculated the coherence scores for our language model while varying the number of topics generated by the model from 2 to 40 topics. From this analysis (Figure 4), we found that the coherence score for our model in this case study peaks at around 12 topics, beyond which the performance of the model is imperceptible and generally leads to loss of coherence between topics. The 12 topics of research generated by the NLP Engine were presented using the **Visualizer**.

The labeled topics and the top ten words clustered under these topics are presented in Table 2. Our results revealed that a large proportion of conservation‐associated research is centered around conservation policy, followed by landscape ecology, vegetation management, and other topics. Such a result gives the researcher an intuitive understanding of what abstracts and topics of research they might be interested in focusing on for further research and analysis.

**TABLE 2:** The 12 Topics generated by the LDA model, labeled using open coding (Khandkar, 2009)

Topic 1 Conservation Policy	Topic 2 Landscape Ecology	Topic 3 Vegetation Management	Topic 4 Forest Ecology	Topic 5 Ecological Methods	Topic 6 Biodiversity Protection
Biodiversity	Habitat	Site	Forest	Datum	Conservation
Conservation	Landscape	Vegetation	Tree	Model	Protect
Management	Richness	Plant	Composition	Base	Biodiversity
Research	Diversity	Grassland	Stand	Distribution	Threaten
Knowledge	Scale	Cover	Density	Method	Reserve
Approach	Factor	Type	Fragment	Information	Threat
Policy	Abundance	Management	Disturbance	Estimate	Include
Process	Pattern	Community	Plantation	Analysis	Area
Develop	Variable	Composition	Ant	Predict	Identify
Resource	Influence	Diversity	Edge	Result	Priority

Topic 7 Climate Change	Topic 8 Population Dynamics	Topic 9 Species Diversity	Topic 10 Humans and Wildlife	Topic 11 Plant Diversity	Topic 12 Aquatic Ecology
Change	Population	Number	Humans	Plant	Community
Increase	Bird	Diversity	Land	Distribution	Soil
Impact	Habitat	Sample	Local	Genetic	Ecosystem
Time	Island	Total	Activity	Region	Water
Year	Native	Record	Resource	Diversity	River
Effect	Mammal	Taxa	Wildlife	Range	Environment
Decline	Large	Area	Wild	Show	Wetland
Loss	Density	Region	Traditional	Pattern	Fish
Climate change	Individual	Group	Management	Flora	Invertebrate
Seed	Size	Richness	People	Endemic	Coastal

To expand on this case study, we examined how these 12 topics of conservation research varied across biodiversity hotspots. Biodiversity hotspots are areas of extraordinary biodiversity and yet are under tremendous anthropogenic pressures of habitat loss and climate change (Myers et al., 2000; Newbold et al., 2015). Obtaining an understanding of distribution of conservation research topics across hotspots can inform research and associated conservation efforts.

We used the **Scraper** to collect abstracts pertaining to five biodiversity hotspots from Springer, using the search words “Hotspot Name” and “Conservation.” We then analyzed how the 12 global conservation research topics, that were previously identified, varied across five biodiversity hotspots: Western Ghats‐Sri Lanka, East Melanesian Islands, Eastern Afromontane Forests, Eastern Himalayas, and the Tropical Andes (these hot spots were chosen in a random manner for the purposes of this case study).

**FIGURE 5:**
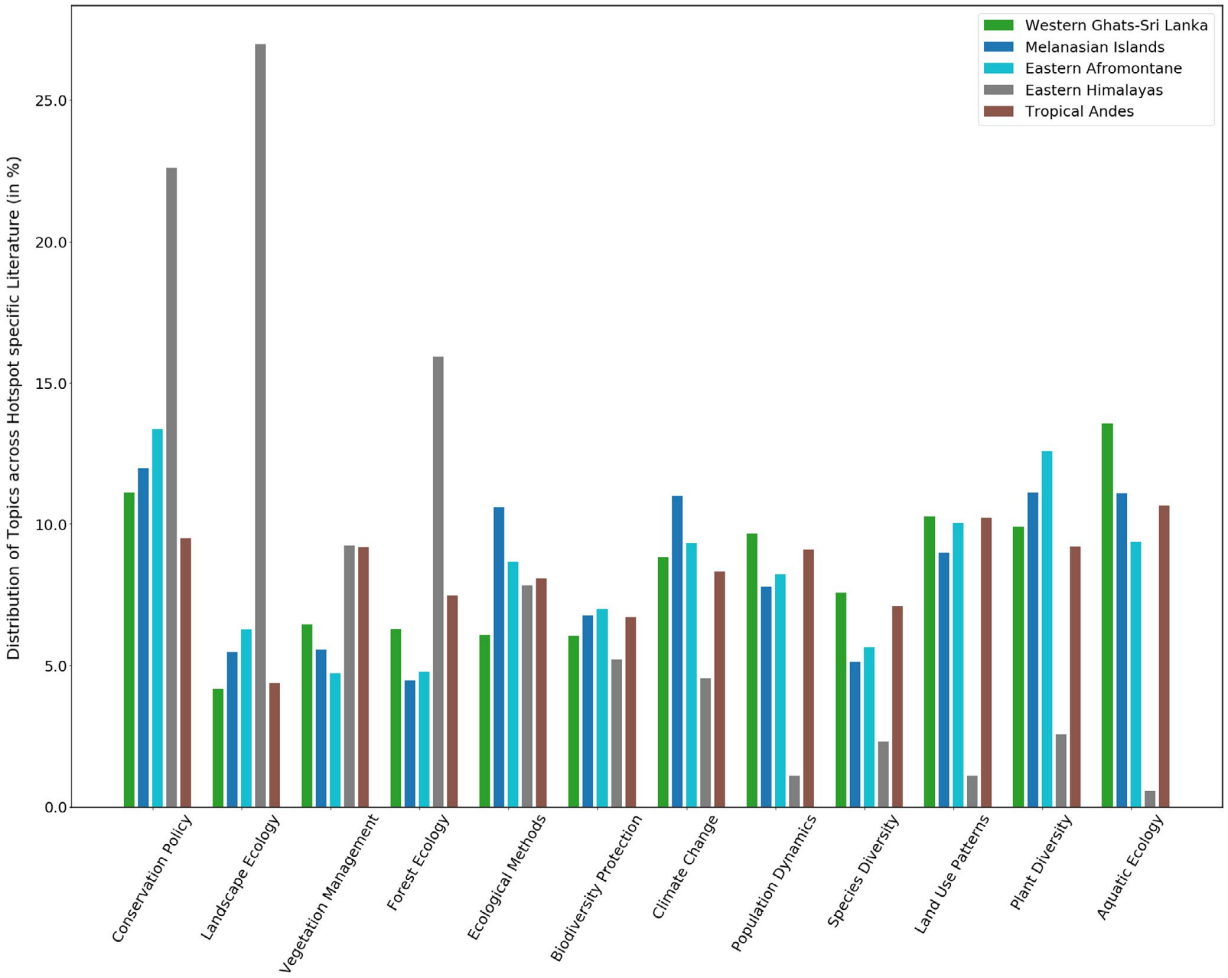
Distribution of global conservation research topics across five biodiversity hotspots

We used the trained LDA model to identify how the 12 global conservation research topics are distributed in the hot spot‐specific literature. This distribution of topics across the five biodiversity hotspots is presented in Figure 5. We arrive at these topic distributions by using getdocument_topics() in gensim.

We observed that the prominent topics of conservation research across the Eastern Himalayas are landscape ecology (26.9%) and conservation policy (22.6%). On the other hand, a majority of conservation‐associated research in the Eastern Afromontane biodiversity hotspot were related to conservation policy (13.3%) and plant diversity (12.6%) (Figure 5).

## 4 COMPARISON WITH EXISTING AUTOMATED CONTENT ANALYSIS TOOLS

A key motivation behind developing *pyResearchInsights* is to provide researchers an open‐source package that performs automated content analysis (ACA). Existing tools and packages are often either pay‐walled or only offer a smaller set of functions. *pyResearchInsights* is the first open‐source end‐to‐end tool that offers a wide range of functions which can either be used for independent analyses or as part of the analysis showcased by the tool. To further illustrate the benefits of this tool over existing ACA tools, we compare several parameters of our tool with those present in existing tools. To do so, we carried out a thorough search of existing R and Python packages and showcased those tools that are comparable to *pyResearchInsights* in their utilities and those tools that are often used in the field of natural language processing.

The criteria for comparison of these existing packages with *pyResearchInsights* included the following: (a) end‐to‐end capability to collect and analyze scientific abstracts. Existing packages are fairly limited in their scraping capabilities, collecting publications from university repositories, or requiring periodic subscriptions. (b) Closed or open‐source availability of codebase. The availability of the codebase dictates whether users can change package settings and tailor process parameters to their liking. (c) Modularity of the package that would allow users to use specific functions for their use cases, instead of using the package as a whole. Through our search for existing tools that provide end‐to‐end analysis of scientific publications, we came across none that offered the end‐to‐end capability of *pyResearchInsights*, coupled with its open‐source codebase and modular functionality. Therefore, we compared *pyResearchInsights* to packages that offer some of these functionalities, either partially or fully in some cases.

As evident from Table 3, there are few tools that provide a complete end‐to‐end solution to the problem of analyzing large volumes of scientific texts. While a few packages offer some of the functionalities of *pyResearchInsights*, they lack other features. For example, packages such as *Paperai* (Mazetti, 2020) and *EDA‐NLP* (Bonhart, 2020) offer comprehensive scraping and visualization capabilities to users but lack a thorough cleaner module, because of which their visualizations are not completely representative of the input corpus of text. In contrast, the Cleaner and Analyzer modules of *pyResearchInsights* ensure commonly used stop words and symbols are weeded out of the corpus, prior to visualization.

**TABLE 3:** Comparison of features of *pyResearchInsights* with those of existing open‐source and pay‐walled ACA tools: While plenty of packages offer some of the features of *pyResearchInsights*, there are none that are completely modular, open‐source and analyze scientific abstracts in an end‐to‐end manner

Package/Tool	Environment	Modular	Open‐source	End‐to‐End Analysis	Jupyter Notebook Support	Google Colab Support	Pricing Model
*ACA*	R	✓	✓	x	✓	✓	Free
*quanteda*	R	✓	✓	Δ	✓	✓	Free
*topicmodels*	R	✓	✓	Δ	✓	✓	Free
*tm*	R	x	✓	x	✓	✓	Free
*openNLP*	R	✓	✓	Δ	✓	✓	Free
*R. TeMiS*	R	✓	✓	Δ	✓	✓	Free
*litsearchr*	R	✓	✓	Δ	✓	✓	Free
*EDA‐NLP*	Python	x	✓	x	✓	✓	Free
*Paperai*	Python	x	✓	✓	✓	✓	Free
*Leximancer*	**‐**	x	x	Δ	x	x	Subscription
*pyResearchInsights*	Python	✓	✓	✓	✓	✓	Free

Note

The Δ symbol implies partial functionality of a given feature or parameter. End‐to‐end here implies the ability of a single package to collect texts from repositories, clean, analyze, and present their topic modeling results.

Packages such as *tm* (Feinerer et al., 2008), *openNLP* (Hornik, 2014), and *R. TeMiS* (Bouchet‐Valat & Bastin, 2018) provide users with extensive text mining capabilities such as tokenization, clustering, and analysis, but lack the interactive visualization techniques available in *pyResearchInsights*. These visualizations allow even novice users to explore and infer areas of research generated from the text files supplied. Such visualization techniques are lacking in *quanteda* (Benoit et al., 2018) as well, which allows users to clean, tokenize, and present only the temporal frequency of words in the corpus, similar in functionality to the Cleaner and Analyzer modules of our package, but lack the interactive pyLDAvis visualizations offered by *pyResearchInsights*’ NLP Engine and Visualizer. A package like *litsearchr* (Grames et al., 2019), on the other hand, offers the user a reproducible search strategy using text‐mining and word co‐occurrence networks to identify important terms to include in a search strategy, when conducting a systematic literature review. While *litsearchr* is particularly useful, it lacks a scraper module that can access scientific repositories such as *pyResearchInsights* and is limited to scraping only open‐access thesis repositories.

Pay‐walled tools such as Leximancer (Smith & Humphreys, 2006) have robust cleaning and visualization capabilities but lack abstract retrieval modules. Furthermore, such tools are available on a subscription basis, charging individual users approximately $560 per year, thereby making it inaccessible to researchers from less endowed programs. Given that these tools are closed‐source, users are limited by the kind of environments that they can be run on. A key advantage of open‐source R and Python packages is the ability to run them on Google Colab and Jupyter Notebooks, leveraging freely available powerful cloud computing for analysis, which is lacking in the case of closed‐source packages.

Although we compared features of *pyResearchInsights* with those of existing tools, it is important to note that our tool has its own set of limitations, despite offering a wide range of utilities. For example, we currently lack settings to fine‐tune the parameters of the content analysis process, such as pruning the dataset of duplicate texts and identifying and merging of synonymic words encountered during the training process. These settings prevent topic models from being trained on duplicate texts that are cluttered with repeated words and ensure that the topic models are representative of the texts provided by the user. While these options do not yet appear in our package, our open‐source alternative offers comparable features that exist in other tools, pay‐walled or otherwise.

## 5 CONCLUSIONS

*pyResearchInsights* is meant to be used as an aid to traditional literature survey techniques, carried out during the exploratory stages of research. The topic modeling results generated by the package should not be cited as conclusive proof of the significance or insignificance of a particular topic, relative to other topics in the literature. Lastly, the language used in scientific publications is linguistically nuanced, and hence, the results generated by the package should be interpreted accordingly.

## CONFLICT OF INTEREST

None declared.

## AUTHOR CONTRIBUTIONS

**Sarthak J. Shetty:** Data curation (lead); formal analysis (lead); investigation (lead); methodology (lead); writing–original draft (lead); writing–review and editing (equal). **Vijay Ramesh:** Conceptualization (lead); methodology (equal); project administration (lead); supervision (lead); writing–original draft (supporting); writing–review and editing (equal).

### OPEN RESEARCH BADGES

This article has been awarded <Open Materials, Open Data, Preregistered Research Designs> Badges. All materials and data are publicly accessible via the Open Science Framework at URL: https://github.com/SarthakJShetty/pyResearchInsights; https://zenodo.org/record/4518917#.YSjkp45KhPY; Codebook: https://colab.research.google.com/drive/1gZjOKr5pfwVMuxCSaGYw20ldFpV4gVws?usp=sharing.

## Data Availability

The topic modeling visualizations presented in each case study are generated as HTML files and can be viewed in a web browser. These visualizations can be found here: https://doi.org/10.6084/m9.figshare.13637171. The retrieval of publications and generation of topic modeling results presented in Case Study A were carried out end‐to‐end on a Google Colab Notebook that can be viewed here: https://colab.research.google.com/drive/1gZjOKr5pfwVMuxCSaGYw20ldFpV4gVws?usp=sharing.
